# Metagenomic shotgun sequencing of blood to identify bacteria and viruses in leukemic febrile neutropenia

**DOI:** 10.1371/journal.pone.0269405

**Published:** 2022-06-16

**Authors:** Prakhar Vijayvargiya, Adeline Feri, Mathilde Mairey, Cécile Rouillon, Patricio R. Jeraldo, Zerelda Esquer Garrigos, Matthew J. Thoendel, Kerryl E. Greenwood-Quaintance, M. Rizwan Sohail, Priya Sampathkumar, Megan T. Spychalla, A. K. Stewart, Mrinal M. Patnaik, Aaron J. Tande, Stéphane Cruveiller, Irene Hannet, Pascale Beurdeley, Robin Patel

**Affiliations:** 1 Division of Infectious Diseases, Mayo Clinic College of Medicine and Science, Rochester, Minnesota, United States of America; 2 Division of Infectious Diseases, University of Mississippi Medical Center, Jackson, Mississippi, United States of America; 3 PathoQuest SAS, Paris, France; 4 Department of Surgery, Mayo Clinic College of Medicine and Science, Rochester, Minnesota, United States of America; 5 Center for Individualized Medicine, Mayo Clinic College of Medicine and Science, Rochester, Minnesota, United States of America; 6 Division of Clinical Microbiology, Mayo Clinic College of Medicine and Science, Rochester, Minnesota, United States of America; 7 Division of Hematology, Mayo Clinic College of Medicine and Science, Rochester, Minnesota, United States of America; 8 Division of Hematology/Oncology, Mayo Clinic College of Medicine and Science, Scottsdale, Arizona, United States of America; University of Illinois College of Medicine, UNITED STATES

## Abstract

Despite diagnostic advances in microbiology, the etiology of neutropenic fever remains elusive in most cases. In this study, we evaluated the utility of a metagenomic shotgun sequencing based assay for detection of bacteria and viruses in blood samples of patients with febrile neutropenia. We prospectively enrolled 20 acute leukemia patients and obtained blood from these patients at three time points: 1) anytime from onset of neutropenia until before development of neutropenic fever, 2) within 24 hours of onset of neutropenic fever, 3) 5–7 days after onset of neutropenic fever. Blood samples underwent sample preparation, sequencing and analysis using the iDTECT® Dx Blood v1® platform (PathoQuest, Paris, France). Clinically relevant viruses or bacteria were detected in three cases each by metagenomic shotgun sequencing and blood cultures, albeit with no concordance between the two. Further optimization of sample preparation methods and sequencing platforms is needed before widespread adoption of this technology into clinical practice.

## Introduction

Infectious complications of neutropenia occur in more than 80% of patients who undergo chemotherapy for hematological malignancy and 10–50% of those with visceral malignancies [1]. In most cases, febrile neutropenia is treated with empiric antibiotics without knowledge of the underlying pathogen. In the antibiotic resistance era, pathogen identification and target-directed therapy rather than empiric therapy should be considered.

The microbiology of febrile neutropenia has changed over time [2–4]. Bacteria are the most commonly detected microorganisms (10–25%), followed by fungi (4%), with viruses being least commonly documented [1]. Because of their risk of a broad range of infections, patients with neutropenic fever undergo extensive workup that, in addition to blood cultures, often includes urine culture, computed tomography of chest, abdomen, pelvis and sinuses, serum aspergillus antigen, serum (1,3)-β-D-glucan, and/or gastrointestinal and respiratory pathogen panel. Nevertheless, a specific infectious etiology is only documented in 20–30% of febrile episodes with current diagnostic approaches [1]. Blood cultures have limited yield because they only detect easily culturable organisms (thus omitting nonculturable or fastidious bacteria, most fungi, and viruses) and because of the almost universal use of antibiotic prophylaxis in this population. In some cases, neutropenic fever may be a manifestation of a non-infectious syndrome [5], including underlying malignancy, chemotherapy, thrombosis and other causes. In a study of 123 patients with neutropenic fever, a virus was detected by quantitative real-time polymerase chain reaction (PCR) in 42% of samples, suggesting that novel technologies that sensitively detect microorganisms might be useful to reveal the etiology of febrile episodes in cases where fever is a symptom of an infectious syndrome [6–8].

Metagenomic shotgun sequencing is a blanket sequencing technique that has the potential to analyze all genetic material present in a sample. Over the last few years, metagenomic shotgun sequencing has been applied in clinical practice, showing promising results and enabling detection of microorganisms not detected by conventional tests and even previously unrecognized human pathogens [9–12]. Clinical studies to date have primarily focused on central nervous system infections, periprosthetic joint infections, sepsis and infections in immunocompromised hosts [9, 13–15]. Heterogeneity of the types of immunocompromised hosts studied limits generalization of results to patients with febrile neutropenia. PathoQuest (Paris, France) is developing a proprietary metagenomic shotgun sequencing platform (iDTECT^®^ Dx Blood v1), for febrile neutropenia patients; the approach restricts analysis to genetic materials from intact microorganisms, excluding cell-free DNA.

The goal of this study was to evaluate the clinical utility of metagenomic shotgun sequencing of blood in patients with acute leukemia and concomitant febrile neutropenia and to attempt to shed light on hitherto unidentified causes of neutropenic fever using the PathoQuest assay.

## Methods

### Study population

This study was approved by the Mayo Clinic Institutional Review Board (ID: 17–003217). We prospectively enrolled subjects with acute myeloid and acute lymphoblastic leukemia by obtaining written informed consent. Adult subjects (≥18 years of age) scheduled to receive chemotherapy and expected to develop neutropenia were included. Subjects with acute promyelocytic leukemia, hematopoietic stem cell transplantation or on intravenous antibiotics for over 24 hours were excluded.

### Specimens and sample collection

**Fig 1:**
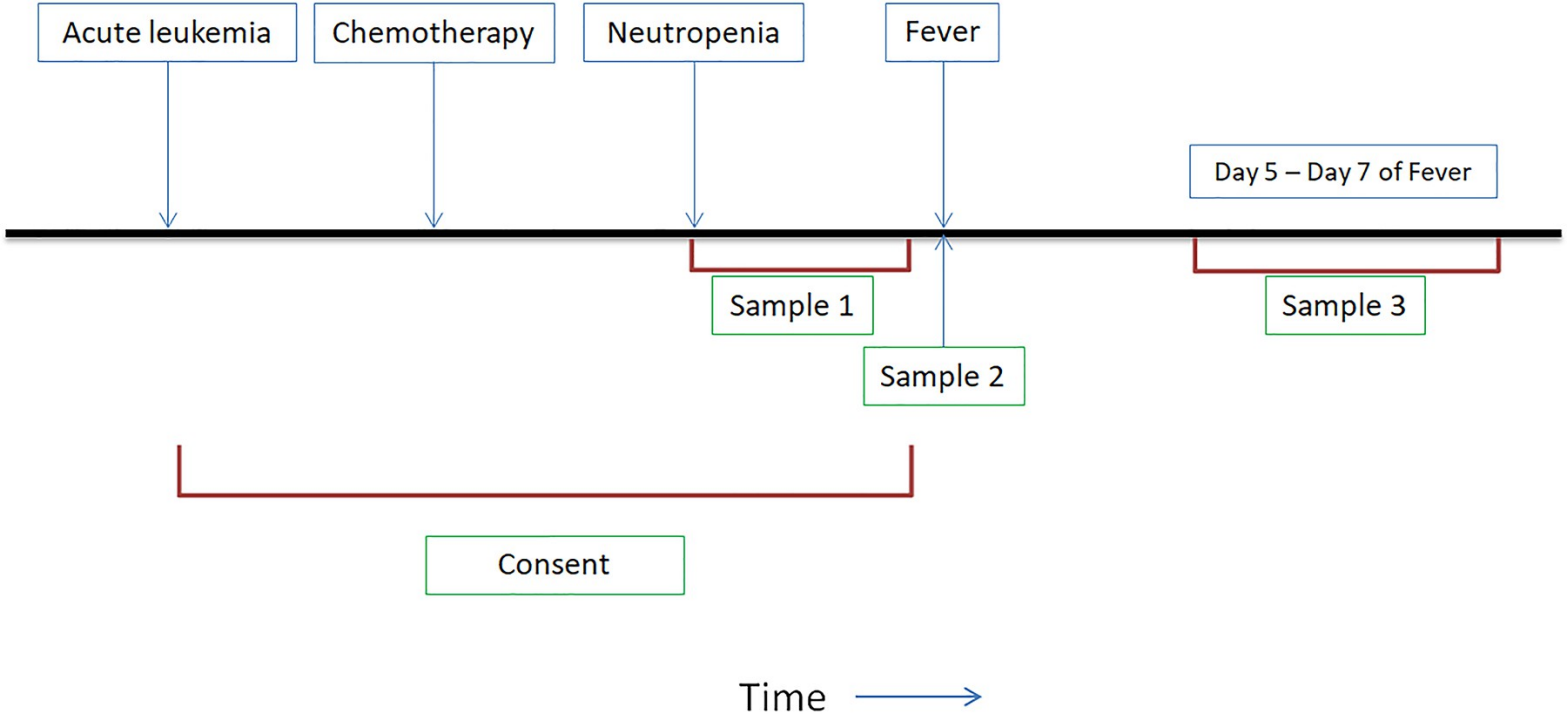
Timeline of blood sample collection for metagenomic shotgun sequencing.

Whole blood (5 ml) was collected in EDTA tubes (Becton, Dickinson and Company, Franklin Lakes, NJ) from each subject at three time points: 1) anytime from the onset of neutropenia until before development of neutropenic fever, 2) within 24 hours of onset of neutropenic fever, and 3) 5–7 days after onset of neutropenic fever (Fig 1). Enrolled subjects were included if sample 2 was available (even if sample 1 and/or sample 3 were not available).

### Sample preparation and analysis

Sample preparation and nucleic acid extraction were performed within four days of collection and according to the iDTECT^®^ Dx Blood v1 instructions for use. Details regarding definitions, sample preparation, sequencing and analysis are available in the S1 File.

Each subject’s electronic medical record was retrospectively reviewed. Information regarding microbiological data, cause of neutropenic fever, antibiotic prophylaxis, and treatment, were collected. If an infectious agent was identified by standard microbiological tests, it was clinically adjudicated to determine clinical significance. With adjudication, the inference of the clinical infectious disease team involved in patient care was considered. In addition to infectious causes of neutropenic fever, potential noninfectious causes were noted. The final classification of infectious *versus* non-infectious cause of fever was based on assessment of the hematology and infectious diseases teams involved in direct patient care (chart review) and clinical adjudication by the study team (retrospective review).

The team responsible for analysis of metagenomic shotgun sequencing results was blinded to the subject characteristics or microorganisms identified by standard microbiological tests. Once the metagenomic shotgun sequencing results were available, these were compared to available clinical data to assess relevance of results. Clinically significant pathogens identified by metagenomic shotgun sequencing were noted as clinically relevant viruses or bacteria (CRVB). Results of metagenomic shotgun sequencing were not revealed to the patients or healthcare providers involved in the patients’ direct care.

## Results

### Subject classification and characteristics

A total of 142 subjects with acute leukemia were approached between July 2018 and September 2019. Of these, 83 consented to be enrolled in the study. A blood sample collected within 24 hours of onset of intravenous antibiotics (sample 2) was available from 20 subjects; these 20 were included in the final analysis. The rest of the subjects either did not develop a fever or had been on intravenous antibiotics for more than 24 hours before sample 2 could be collected. Subject characteristics are shown in Table 1.

**Table 1:** Baseline characteristics of the study cohort (n = 20).

Characteristic	Subject, No. (%)^a^
Sex, female	8 (40)
Age, median (IQR), y	55.5 (40–69)
Type of leukemia	
Acute myeloid leukemia	16 (80)
Acute lymphoid leukemia	4 (20)
Chemotherapy cycle	
Induction	17 (85)
Consolidation	3 (15)
Prophylaxis	
Levofloxacin	16 (80)
Cefdinir	3 (15)
Cefadroxil	1 (5)
Amoxicillin/clavulanate	1 (5)
Trimethoprim/sulfamethoxazole	2 (10)
Acyclovir	20 (100)
Posaconazole	10 (50)
Caspofungin	11 (55)
Duration of fever, median (IQR), d	3 (1–5)
Duration of neutropenia until fever, median (IQR), d	5.5 (0–15)
Samples available for sequencing	47
Sample 1	11 (55)
Sample 2	20 (100)
Sample 3	16 (80)
Sample 2 collection	
Before antibiotic administration	14 (70)
After antibiotic administration, but within 24 hours of fever onset	6 (30)
Treatment of neutropenic fever	
Cefepime	16 (80)
Piperacillin/tazobactam	6 (30)
Meropenem	1 (5)
Oseltamivir	1 (5)
Vancomycin	13 (65)
Actively receiving chemotherapy at the time of fever	6 (30)
Duration of treatment with antibiotics, median (IQR), d	7 (5–13)
Duration of treatment for subjects where infection was not suspected (n = 10), median (IQR), d	5 (4–7)
Duration of treatment for subjects with suspicion for infection (n = 10), median (IQR), d	12 (9–15)

Abbreviations: IQR, interquartile range.

^a^Data are shown as number (%) unless otherwise indicated

### Comparison of metagenomic shotgun sequencing to blood cultures

Blood cultures grew a bacterium in three cases: *Streptococcus mitis* group in two and *Leptotrichia* species in one (Table 2). There was presence of CRVB in three cases by metagenomic shotgun sequencing: rhinovirus in a subject with respiratory tract infection, *Staphylococcus aureus* in a subject with peri-anal cellulitis and *Staphylococcus epidermidis* in a subject with a potential bloodstream infection. Results of blood culture and metagenomic shotgun sequencing were discordant in all cases. Of the two *S*. *mitis* group bacteremia cases, one had only one out of nine bottles positive for the organism, suggesting low-grade transient bacteremia or even a contaminant. In the other two cases with positive blood cultures (*S*. *mitis* group and *Leptotrichia* species), subjects had been started on antibiotics before the sample for metagenomic shotgun sequencing was collected. GB virus was identified by metagenomic shotgun sequencing in two cases, neither of whom had hepatitis; this significance of this virus in these subjects was considered unknown. Simian virus 40, a pathogen with oncogenic potential, was identified by metagenomic shotgun sequencing in a patient with bloodstream infection; the subject did not have a history of polio virus vaccine. Overall performance of metagenomic shotgun sequencing *versus* blood culture is shown in S1 Table.

**Table 2:** Metagenomic shotgun sequencing *versus* blood cultures and standard microbiological results.

No	Blood culture	Metagenomic shotgun sequencing	Standard tests (specimen type)	Duration of antimicrobial therapy (days)	Type of Leukemia	New or relapsed	Chemotherapy cycle	Etiology of fever based on medical record review
1	None	None	None	2	ALL	New	Consolidation	Deep venous thrombosis
2	None	None	None	5	AML	Relapsed	Induction	Chemotherapy related fever
3	None	None (GB virus*)	None	11	AML	New	Induction	Colitis/pulmonary nodules
4	None	None	None	21	AML	New	Induction	Non-ST elevation myocardial infarction/acute diverticulitis
5	None	None	None	5	ALL	New	Induction	Chemotherapy related fever
6	None	Rhinovirus	Rhinovirus (nasopharyngeal swab)	13	AML	New	Induction	Respiratory infection
7	None	None	None	4	AML	New	Induction	Chemotherapy related fever
8	None	None	None	4	AML	New	Induction	Pneumonia
9^†^	None	None	None	7	AML	New	Induction	Chemotherapy related fever
10	None	None	*Pseudomonas aeruginosa* (sputum)	14	AML	Relapsed	Induction	*Pseudomonas* pneumonia
11^†^	*S*. *mitis* group (2/2 sets)	None	*S*. *mitis* group (blood culture)	14	AML	New	Consolidation	*S*. *mitis* bloodstream infection
12^†^	*Leptotrichia wadei* (1/2 sets) and *Leptotrichia buccalis* (1/2 sets)^‡^	*S*. *epidermidis* (also Simian virus 40)	*L*. *wadei* and *L*. *buccalis* (blood culture)	17	AML	New	Induction	Mucositis with potential transient bacteremia
13	None	*S*. *aureus*	None	10	ALL	Relapsed	Induction	Peri-anal cellulitis
14	*S*. *mitis* (1/3 sets; 1/9 bottles)	None	*S*. *mitis* group (blood culture)	7	AML	New	Induction	Transient bacteremia or contaminant
15^†^	None	None	None	7	AML	New	Induction	Superior vena cava thrombosis
16^†^	None	None (GB virus*)	Influenza A and respiratory syncytial virus (nasopharyngeal swab)	10	ALL	Relapsed	Consolidation	Upper respiratory infection
17	None	None	None	3	AML	Relapsed	Induction	Chemotherapy related fever
18^†^	None	None	None	7	AML	New	Induction	Drug fever with rash
19	None	None	None	5	AML	Recurrent	Induction	Chemotherapy related fever
20	None	None	None	10	AML	New	Induction	Suspected drug reaction

Abbreviations: AML, acute myeloid leukemia; ALL, acute lymphoid leukemia.

*Clinical significance of GB virus is unclear.

^†^ Samples where antibiotics were administered prior to sample collection for metagenomic shotgun sequencing.

^‡^ *Leptotrichia wadei* and *Leptotrichia buccalis* each grew from a different set of blood cultures

### Comparison of metagenomic shotgun sequencing to standard microbiological tests

Besides blood culture results, as noted above, three other subjects had positive microbiological laboratory tests, all from the respiratory tract. These included, respectively, rhinovirus and influenza A/respiratory syncytial virus infections in two subjects with upper respiratory tract infection, and a sputum culture for *Pseudomonas aeruginosa* in a subject with pneumonia. Overall performance of metagenomic shotgun sequencing *versus* standard tests is shown in S2 Table.

### Noninfectious cause of fever

In 13 cases, no pathogen was identified by metagenomic shotgun sequencing or standard microbiological tests. In 10 of these cases, thrombosis (2), chemotherapy (6), or drug reactions (2) were considered probable etiologies of fever, with no infectious syndrome identified. The duration of antimicrobial therapy for these 10 patients was shorter than among patients in whom infection was either identified or suspected (median of 5 *versus* 12 days). Additional clinical information is available in S3 Table.

### Sample 1 and sample 3

None of the samples collected before or 5–7 days after onset of fever had a CRVB detected by metagenomic shotgun sequencing.

## Discussion

Despite significant advances in clinical microbiology testing, the etiology of neutropenic fever remains elusive in most cases. This study aimed to use metagenomic shotgun sequencing to detect pathogen(s) in patients with neutropenic fever. Of 20 cases enrolled in the study, 13 had no evidence of infection by metagenomic shotgun sequencing or with standard tests, consistent with the concept of neutropenic fever being of noninfectious origin in many cases. Metagenomic shotgun sequencing detected a potential pathogen that was not detected by blood cultures in two cases (detected by conventional microbiological tests in one case) suggesting that metagenomic shotgun sequencing may be a supplemental test to determine the etiology of some cases of neutropenic fever. When results from all available tests were analyzed together, a potential etiological agent for neutropenia fever was identified in 7/20 (35%) cases overall, and 7/10 (70%) cases of suspected infection.

Metagenomic shotgun sequencing has been touted as a revolutionary approach with the potential to change the landscape of microbiology testing. Over the last decade, there has been exponential growth of interest in development of protocols for shotgun metagenomic sequencing. Clinical adoption of these tests is, however, hampered by lack of clarity surrounding utility in clinical scenarios, difficulty with result interpretation, high cost, and long turnaround time.

Gyarmati et al. demonstrated, in nine patients with acute leukemia, that shotgun metagenomic sequencing identified viruses or fungi in the blood of patients with neutropenic fever [16]. Their study aimed at assessing the microbial content of blood in neutropenic patients; therefore, clinical adjudication was not performed to establish a causal association with neutropenic fever. In two of nine cases, there was no microbial DNA detected; three cases had only bacterial DNA detected, while four cases had DNA from bacteria, viruses, and fungi detected. The presence of more than one organism by metagenomic shotgun sequencing can be a strength as well as limitation. Clinical interpretation can be challenging in cases where multiple organisms are detected in the absence of a clinical picture with biologic plausibility supporting the pathogenicity of all detected organisms. The onus then falls on the clinician to determine whether the detected organisms represent a real infection, contamination, colonization, or background noise.

With an aim to reduce background noise, PathoQuest utilizes internal and external controls and an internally validated scoring system to limit the number of reported pathogens. PathoQuest’s assay, iDTECT^®^ Dx Blood v1, is the first IVD CE-marked metagenomic shotgun sequencing-based platform for immunocompromised hosts. This method takes 48 hours from sample collection to results. While Gyarmati et al. developed a process to detect only DNA from blood, iDTECT^®^ Dx Blood v1 combines DNA and RNA extraction from blood to capture RNA viruses as well. In a multicenter, blinded, prospective study, a prototype iDTECT platform was able to identify more CRVB than conventional microbiological methods [36/101 (36%) *versus* 11/101 (11%), respectively; p<0.001]; the study included patients with any immunodeficiency, including autoimmune and pro-inflammatory diseases [9].

In the United States, a microbial cell-free DNA-based metagenomic shotgun sequencing test has been made commercially available by Karius (Redwood City, CA). The Karius assay claims to detect a broad range of pathogens, including DNA viruses, bacteria, fungi, and parasites (but not RNA viruses). This test has been validated in a cohort of 182 patients with clinically adjudicated sepsis, where it yielded a higher detection rate compared to blood cultures and standard microbiological tests (92.9 *versus* 34.6 *versus* 72.5%, respectively). DNA from more than one microorganism was detected in 28.6% of samples [17]. More research is needed to identify high-yield patient populations for metagenomic shotgun sequencing based assays to avoid false-positive results and deliver high-value care. A retrospective evaluation of the Karius test showed that a positive impact result was obtained in 6 of 82 (7.3%) tests, with negative impact in three cases (3.7%) [18].

In the current study, a homogenous population of acute leukemia patients was studied and utility of the iDTECT^®^ Dx Blood v1 test to identify pathogens in blood at the time of neutropenic fever was evaluated. In 3/20 cases, a CRVB was identified that was either not detected or was discordant with conventional microbiological methods. There were also three cases where blood cultures had microbial growth not detected by metagenomic shotgun sequencing. In one subject with *S*. *mitis* group bloodstream infection, the patient had been on antibiotics prior to sample collection for metagenomic shotgun sequencing. Rapid clearance of bacteremia with antibiotics might explain the false-negative sequencing result. In the other *S*. *mitis* group case, negative by metagenomic shotgun sequencing, the bacterium grew in only one of nine concurrently collected blood culture bottles, suggesting that the blood culture growth was either a contaminant or reflected low-grade transient bacteremia. In the third case, also on antibiotics prior to sample collection for sequencing, *Leptotrichia* species were isolated from blood culture while metagenomic shotgun sequencing detected *S*. *epidermidis*. These organisms could be potential etiological agents of febrile neutropenia, with all or some reflecting bacterial translocation from the gastrointestinal tract secondary to mucositis and the *S*. *epidermidis* alternatively reflecting central line-related bloodstream infection. Whether blood samples were collected from peripheral blood draws or from central lines is unknown. It has been suggested that metagenomic shotgun sequencing may be able to predict development of central line-related bloodstream infection; however, in the present study, samples sent for sequencing prior to onset of fever did not reveal pathogens.

Metagenomic shotgun sequencing and microbiological tests were both negative in 13/20 cases; of these, six were actively receiving chemotherapy, two had thrombosis, and two had drug-related fever. Drug fever from administration of chemotherapeutic drugs frequently complicates the management of neutropenic fever, usually developing three or four days after initiation of chemotherapy [5, 19]. Even in the absence of neutropenia, cytarabine administration can cause fever in as many as 43% of cases. As infection cannot be conclusively ruled out, these 13 patients were started on antibiotic therapy for neutropenic fever per institutional protocol; however, extensive microbiological studies and metagenomic shotgun sequencing did not find a pathogen. 14/20 (70%) of patients were newly diagnosed with acute leukemia and, therefore, received shorter durations of cumulative neutropenia compared to patients who received multiple cycles of prior chemotherapy. Theoretically, shorter durations of cumulative neutropenia may translate into lower risks of infection compared to prolonged durations of cumulative neutropenia. These observations, along with a retrospective review of these cases, suggests that clinicians had a low suspicion for infection and felt comfortable stopping antibiotics earlier. In the era of antimicrobial resistance, unnecessary antibiotic treatment of patients can potentially be avoided if the presence of a microbial pathogen can be definitively ruled out. To this end, metagenomic shotgun sequencing-based approaches could be further evaluated as an antimicrobial stewardship tool to deescalate antibiotics to prophylactic regimens.

The current study builds on the hypothesis that in new leukemic patients with early fever after chemotherapy, an infectious agent may not be the offending culprit. However, before results of the study can be incorporated into clinical practice, a larger study is needed to confirm the findings. A limitation of the study is that samples for metagenomic shotgun sequencing and routine microbiological testing were not always collected at the same time; this could potentially introduce bias. A current limitation of the PathoQuest’s platform is that iDTECT^®^ Dx Blood v1 detects only viruses and bacteria, and not fungal organisms, which can be pathogens in immunocompromised populations. A direct comparison of cell-free DNA based approach and sequencing of genomic material extracted from intact microorganisms would be helpful to optimize the sample preparation process. Methods to optimize iDTECT assay are a work in progress and have been included automation of the nucleic extraction phase, replacement of the reverse transcriptase—successive tags for library amplification step by a reverse transcriptase-second strand synthesis phase prior to library preparation, targeted sample preparation with enrichment of pathogen sequences contained in the libraries using pathogen specific capture probes to help identify resistance genes, and automation of library preparation.

In conclusion, metagenomic shotgun sequencing could potentially be used as a supplement to standard tests to increase the yield of microbiological diagnosis. However, improvements in and optimization of sample preparation methods and sequencing platforms will be needed for widespread adoption of this approach into clinical practice.

## Supporting information

S1 File (DOCX)

S1 Table Performance of shotgun metagenomic sequencing *versus* blood culture. (DOCX)

S2 Table Performance of shotgun metagenomic sequencing *versus* standard tests. (DOCX)

S3 Table Clinical characteristics of the study subjects. (DOCX)

S4 Table List of organisms identified in the no template control. (DOCX)

## Abbreviations

PCR: Polymerase chain reaction
DNA: Deoxyribonucleic acid
RNA: Ribonucleic acid
CRVB: Clinically relevant viruses or bacteria
NTC: No template control
AML: Acute myeloid leukemia
ALL: Acute lymphoid leukemia
IQR: Interquartile range
TMP-SMX: Trimethoprim-sulfamethoxazole
