# Supplementary material for: Metagenomic shotgun sequencing of blood to identify bacteria and viruses in leukemic febrile neutropenia
Source: PLoS One. 2022 Jun 16;17(6):e0269405. doi: 10.1371/journal.pone.0269405 (PMC9202879; doi:10.1371/journal.pone.0269405)
Supplement: S1 File — (DOCX) [file pone.0269405.s001.docx]

**Supplementary Information**

**Supplementary Methods**

# Definition

In accordance with guidelines set by the Infectious Diseases Society of America, the following definitions were used: Fever in neutropenic patients was defined as a single oral temperature measurement of >38.3°C or a temperature of >38.0°C sustained over a 1-hour period [1]. Neutropenia was defined as an absolute neutrophil count <500 cells/mm^3^ or an absolute neutrophil count expected to decrease to <500 cells/mm^3^ during the ensuing 48 hours. Bloodstream infection was defined as growth of a known pathogen from one or more blood cultures. For potential skin contaminants (based on the National Healthcare Safety Network common commensal list) growth from two or more blood cultures was required to be considered significant [2].

**Sample preparation and sequencing**

Whole blood samples were aliquoted into two fractions: 1 ml whole blood and 1 ml plasma for bacterial and viral genome detection/identification, respectively, and stored at 4°C until further processing. Samples underwent nucleic acid extraction within four days of collection. Bacterial and viral fractions were spiked with internal controls (DNA phages for bacterial, and DNA and RNA phages for viral fractions) to assess assay quality. Sample preparation and nucleic acid extraction were performed according to iDTECT® Dx Blood v1 instructions for use (PathoQuest, Paris, France). To assess environmental and process-associated contaminants, a no template control (NTC), DEPC-treated sterile DNase/RNase-free water, underwent the same treatment as patient samples. Briefly, viruses were extracted from 150 µL of plasma following pre-treatment with saponin (Sigma Aldrich, Saint Louis, MO) along with two nucleases, benzonase (Merck KGaA, Darmstadt, Germany) and baseline-zero (Euromedex, France) to remove free nucleic acids (from host or degraded pathogens), allowing focused detection of intact viable viruses. Subsequent steps were performed with the IndiSpin Pathogen kit (Indical Bioscience GmbH, Leipzig, Germany) following the manufacturer’s recommendations. Bacterial DNA was extracted from 300 µL of whole blood using the QIAamp DNA Microbiome kit (Qiagen, Hilden, Germany); prior to nucleic acid extraction, cells were lysed, samples centrifuged, pellets resuspended, and free nucleic acids degraded with benzonase and saponin. Bacterial and viral fractions were spiked with internal controls (DNA phages for bacterial, and DNA and RNA phages for viral fractions) to assess quality. Extracted nucleic acids from patient samples and NTC underwent a quasi-linear amplification step. To detect potential viral RNA genomes, a reverse transcription step was performed using the SuperScript III First-Strand Synthesis System (Thermo Fisher Scientific, Waltham, MA) and a single primer harboring a specific 5’-end sequence and 8 consecutives G/T nucleotides (Primer-K8 with K = G or T) at the 3’- end. The Successive Tags for Library Amplification (STLA) method was performed with Vent exo- DNA polymerase (New England Biolabs). A single primer was used (Primer-K8, 5’-GTGAGTGATGGTTGAGGTAGTGTGGAGKKKKKKKK-3’) for reverse transcription for 6 random amplification cycles. From the second cycle, priming on tagged fragments would be expected to lead to hairpin structures harboring the adapter sequence at both extremities. Amplification of viral and bacterial DNA was performed using a primer corresponding to the specific sequence of the primer used during the first-strand cDNA synthesis deleted at the 3’ end (5’-GTGAGTGATGGTTGAGGTAGTGTGGAG-3’). This step exponentially amplified hairpin structures harboring the adapter sequence at both extremities.

Library preparation and sequencing were performed following recommendations from Illumina (San Diego, CA) for Nextera XT library preparation. Sequencing was performed on a NextSeq instrument (Illumina) using the NextSeq 500/550 High Output kit v2.5. Sequencing was single read and read length was 1x150 nucleotides. An average of 20 million reads was generated per sample. Reports were automatically generated by PathoQuest’s bioinformatics pipeline using a curated database of more than 1,600 clinically relevant bacteria and viruses.

Results of metagenomic shotgun sequencing were not revealed to the patients or healthcare providers involved in the patients’ direct care. Microorganisms identified by metagenomic shotgun sequencing were not considered significant if the same organisms were present in the NTC sequenced with the corresponding patient samples.

Blood cultures were performed using the Becton Dickinson BD BACTEC FX™ platform (Becton, Dickinson, and Company, Franklin Lakes, NJ). A typical set consisted of two BD BACTEC™ Plus Aerobic/F bottles and one BD BACTEC™ Lytic Anaerobic/F bottle. Routinely, at least two blood culture sets are drawn per patient.

**Bioinformatics analysis of high-throughput data**

Sequenced blood samples were processed through 3 steps, pre-processing of data, taxonomic assignation, and final validation. During the first step, short reads displaying peculiar characteristics (low bases qualities, read duplicates and reads harboring homopolymers runs) were filtered out to retain high-quality data for subsequent analyses. In parallel, coverage statistics of internal controls were computed. Overall, this step was needed to minimize noise in results delivered at the end of the pipeline. Coverage statistics of internal controls ensure validity of the experiment. High quality reads were then assigned to species by screening them against databases containing representative sequences of pathogens (i.e., bacteria, segmented and non-segmented viruses) using a mapping approach. Resulting alignments were parsed to extract sequencing depth, breadth of coverage and specificity of the reported hit. For this last parameter, three states were considered i) unique/specific hit, ii) hit shared by the genus, or iii) hit shared by several genera.

Based on results delivered in the previous step, a confidence score which is a function of sequencing depth, breadth of coverage and coverage continuity, was computed. Finally, scored entries were filtered according to predefined thresholds (different for bacteria and viruses) to report potentially present organisms.

NTC samples were processed in the same way as patient samples and used to withdraw organisms that might have been sequenced due to their presence in the environment at the time of the experiment.

**Generation of microbial reference database**

The iDTECT database and software has been developed by PathoQuest BioIT engineers and copyrighted. Taxonomic information was downloaded from public databases (NCBI-Taxonomy) and made available to a panel of microbiology experts through a dedicated web application specific for purpose and in which they could review available species and select those that are clinically relevant. Each expert worked independently (i.e., one expert did not know which species had been selected by others) to avoid biases in species selection. Species that received consensus (i.e., selected as clinically relevant) were validated forthright whereas justifications (clinical evidence and publications) for adjudication were requested for others. Once the list was validated, complete or large genomic sequences from selected species were retrieved from public databases (NCBI [US], ENA [European Nucleotide Archive]).

Downloaded sequences were cleaned to maximize size of the representative genomic sequence of an organism while discarding questionable and redundant sequences. Sensitivity validation was performed through *in silico* experiments. This consisted of taking the genomic sequence of one species, splitting it into fragments, screening them against the complete database and evaluating the success rate of finding the species again. This operation was performed for each species in the database.

**Publication of sequences**

Human reads were removed, and raw sequence data for each sample deposited in the Sequence Read Archive (SRA) database at the National Center for Biotechnology Information (NCBI) under BioProject accession number PRJNA674642.

**References**

1. Freifeld AG, Bow EJ, Sepkowitz KA, et al. Clinical practice guideline for the use of antimicrobial agents in neutropenic patients with cancer: 2010 update by the Infectious Diseases Society of America. Clin Infect Dis **2011**; 52(4): e56-93.

2. National Healthcare Safety Network (NHSN) Patient Safety Component Manual. Available at: cdc.gov/nhsn/pdfs/validation/2017/pcsmanual_2017.pdf. Accessed March 2018.the
