# Supplementary material for: Metagenomic shotgun sequencing of blood to identify bacteria and viruses in leukemic febrile neutropenia
Source: PLoS One. 2022 Jun 16;17(6):e0269405. doi: 10.1371/journal.pone.0269405 (PMC9202879; doi:10.1371/journal.pone.0269405)
Supplement: S2 Table — (DOCX) [file pone.0269405.s003.docx]

**Supplemental Table 2.** Performance of shotgun metagenomic sequencing *versus* standard tests

|  | | Standard tests | |  |
| --- | --- | --- | --- | --- |
|  |  | Positive | Negative |  |
| Sequencing | Positive | 1+1 (discordant) | 1 | 3 |
|  | Negative | 4 | 13 | 17 |
|  | Total | 6 | 14 | 20 |
