# Supplementary material for: Metagenomic shotgun sequencing of blood to identify bacteria and viruses in leukemic febrile neutropenia
Source: PLoS One. 2022 Jun 16;17(6):e0269405. doi: 10.1371/journal.pone.0269405 (PMC9202879; doi:10.1371/journal.pone.0269405)
Supplement: S3 Table — (DOCX) [file pone.0269405.s004.docx]

**Supplemental Table 3.** Clinical characteristics of the study subjects.

| **No.** | **Blood culture** | **Metagenomic shotgun sequencing** | **Standard tests (specimen type)** | **Microbiological tests done** | **Etiology of fever based on medical record review** | **Chemotherapy** | **Sample available for sequencing** | **Central line** | **Mucositis** | **GM-CSF** | **ID consult** |
| --- | --- | --- | --- | --- | --- | --- | --- | --- | --- | --- | --- |
| 1 | None | None | None | Blood culture: Negative  CXR: Negative  Urine culture: Negative | Deep venous thrombosis | Cytarabine + Methotrexate + Methylprednisolone | S1, S2, S3 | Yes | Yes | Yes | No |
| 2 | None | None | None | Blood culture:  CXR: Negative  CT chest: Small ground-glass opacification  CT abdomen: Negative  *Brucella* serology: Negative  Q fever serology: Negative  (1→3)-β-D-Glucan assay: Negative  Galactomannan: Negative | Chemotherapy related fever | Idarubicin + Fludarabine + Cytarabine | S1, S2, S3 | Yes | Yes | Yes | Yes |
| 3 | None | None (GB virus) | None | Blood culture: Negative  CXR: Negative  CT chest: Multiple sub-centimeter pulmonary nodules  CT abdomen: Colitis  *Clostridioides difficile* PCR: Negative  (1→3)-β-D-Glucan assay: Negative  Galactomannan: Negative | Colitis/pulmonary nodules | Mitoxantrone + Cytarabine + Etoposide | S1, S2, S3 | Yes | Yes | No | No |
| 4 | None | None | None | Blood culture: Negative  CXR: Subtle opacities in right mid lung  CT chest: Negative  CT abdomen: Acute sigmoid colon diverticulitis  *C. difficile* PCR: Negative | Non-ST elevation myocardial infarction/acute diverticulitis | Cytarabine + Daunorubicin + clinical trial drug | S1, S2, S3 | Yes | No | No | Yes |
| 5 | None | None | None | Blood culture: Negative  CXR: Negative  *C. difficile* PCR: Negative | Chemotherapy related fever | Dasatinib + Hyper CVAD | S1, S2, S3 | Yes | Yes | Yes | No |
| 6 | None | Rhinovirus | Rhinovirus (nasopharyngeal swab) | Blood culture: Negative  CXR: Negative  CT chest: Negative  CT abdomen: Negative  *C. difficile* PCR: Negative  Respiratory pathogen panel: Rhinovirus  Gastrointestinal panel: Negative  Serology for Q fever, and *Bartonella* and *Histoplasma* species: Negative | Respiratory infection | Idarubicin + Cytarabine + Midostaurin | S1, S2, S3 | Yes | No | No | Yes |
| 7 | None | None | None | Blood culture: Negative  CXR: Negative  CT abdomen: Negative | Chemotherapy related fever | Idarubicin + Cytarabine | S1, S2, S3 | Yes | No | No | No |
| 8 | None | None | None | Blood culture: Negative  CXR: Bilateral interstitial infiltrates  CT chest: Bilateral interstitial infiltrates  Urine culture: Negative  (1→3)-β-D-Glucan assay: Negative  Galactomannan: Negative | Pneumonia | Idarubicin + Cytarabine followed by Venetoclax and Decitabine | S1, S2 | Yes | Yes | No | No |
| 9 | None | None | None | Blood culture: Negative  CXR: Negative  *C. difficile* PCR: Negative  Respiratory pathogen panel: Negative | Chemotherapy related fever | Idarubicin + Cytarabine + clinical trial drug | S2, S3 | Yes | No | No | No |
| 10 | None | None | *Pseudomonas aeruginosa* (sputum) | Blood culture: Negative  CXR: Consolidation  CT chest: Pneumonia  Sputum culture: *Pseudomonas aeruginosa* | *Pseudomonas* pneumonia | Idarubicin + Fludarabine + Cytarabine | S2 | No | No | No | Yes |
| 11 | *Streptococcus mitis* group (2/2 sets) | None | *S. mitis* group (blood culture) | Blood culture: *S. mitis* group  CXR: Negative  CT abdomen: Negative  *C. difficile* PCR: Negative  Respiratory pathogen panel: Negative | *S. mitis* bloodstream infection | Cytarabine + Midostaurin | S2, S3 | Yes | No | No | Yes |
| 12 | *Leptotrichia wadei* (1/2 sets) and *Leptotrichia buccalis* (1/2 sets)^‡^ | *S. epidermidis* (also Simian virus 40) | *L. wadei* and *L. buccalis* (blood culture) | Blood culture: *L. wadei* and *L. buccalis*  CXR: Negative | Mucositis with potential transient bacteremia | Idarubicin + Cytarabine | S2, S3 | No | Yes | No | Yes |
| 13 | None | *S. aureus* | None | Blood culture: Negative  CXR: Negative | Peri-anal cellulitis | Idarubicin + Cytarabine | S1, S2, S3 | Yes | No | Yes | Yes |
| 14 | *S. mitis* (1/3 sets; 1/9 bottles) | None | *S. mitis* group (blood culture) | Blood culture: *S. mitis* group  CXR: Negative  CT chest: Multifocal nodular consolidation  CT abdomen: Negative  Urine culture: Negative  *C. difficile* PCR: Negative | Transient bacteremia or contaminant | Cytarabine + Daunorubicin | S1, S2, S3 | Yes | No | No | Yes |
| 15 | None | None | None | Blood culture: Negative  CXR: Negative  *C. difficile* PCR: Negative | Superior vena cava thrombosis | Idarubicin + Cytarabine | S2 | Yes | No | No | No |
| 16 | None | None (GB virus) | Influenza A and respiratory syncytial virus (nasopharyngeal swab) | Blood culture: Negative  CT chest: opacity in right middle lobe  Nasal swab: Influenza/ respiratory syncytial virus  Sputum culture: Negative | Upper respiratory infection | Idarubicin + Cytarabine | S2 | No | No | Yes | No |
| 17 | None | None | None | Blood culture: Negative  CXR: Negative | Chemotherapy related fever | Idarubicin + Cytarabine | S2, S3 | Yes | No | No | Yes |
| 18 | None | None | None | Blood culture: Negative  CXR: Negative  *C. difficile* PCR: Negative | Drug fever with rash | Idarubicin + Cytarabine | S1, S2, S3 | Yes | Yes | No | No |
| 19 | None | None | None | Blood culture: Negative  CXR: Negative | Chemotherapy related fever | Cladribine + Cytarabine + G-CSF + Mitoxantrone | S2, S3 | Yes | No | Yes | No |
| 20 | None | None | None | Blood culture: Negative  CXR: Negative  Urine culture: Negative | Suspected drug reaction | Idarubicin + Cytarabine + Nilotinib | S2, S3 | Yes | Yes | No | No |

**Abbreviations:** CXR, chest x-ray; CT, computed tomography; GM-CSF, granulocyte-macrophage colony-stimulating factor.
