# Supplementary material for: Metagenomic shotgun sequencing of blood to identify bacteria and viruses in leukemic febrile neutropenia
Source: PLoS One. 2022 Jun 16;17(6):e0269405. doi: 10.1371/journal.pone.0269405 (PMC9202879; doi:10.1371/journal.pone.0269405)
Supplement: S4 Table — (DOCX) [file pone.0269405.s005.docx]

Supplemental Table 4. List of organisms identified in the No Template Control.

| *Acetobacter pasteurianus* |
| --- |
| *Acetobacter senegalensis* |
| *Bacillus subtilis* |
| *Lysinibacillus fusiformis* |
| *Bifidobacterium thermophilum* |
| *Bradyrhizobium icense* |
| *Clostridium butyricum* |
| *Clostridium carboxidivorans* |
| *Clostridium scatologenes* |
| *Acidovorax ebreus* |
| *Corynebacterium glutamicum* |
| *Corynebacterium kroppenstedtii* |
| *Corynebacterium vitaeruminis* |
| *Enterococcus cecorum* |
| *Enterococcus hirae* |
| *Lactobacillus buchneri* |
| *Lactobacillus casei* |
| *Lactobacillus paracasei* |
| *Lactobacillus plantarum* |
| *Acinetobacter guillouiae* |
| *Acinetobacter junii* |
| *Moraxella osloensis* |
| *Propionibacterium acnes* |
| *Pseudomonas veronii* |
| *Staphylococcus warneri* |
| *Lactococcus lactis* |
| *Streptococcus mitis* |
| *Streptococcus oralis* |
| *Streptococcus salivarius* |
| *Streptococcus thermophilus* |
| *Betapapillomavirus 2* |
